# The Invasive Mechanism and Impact of *Arundo donax,* One of the World’s 100 Worst Invasive Alien Species

**DOI:** 10.3390/plants14142175

**Published:** 2025-07-14

**Authors:** Hisashi Kato-Noguchi, Midori Kato

**Affiliations:** Department of Applied Biological Science, Faculty of Agriculture, Kagawa University, Miki 761-0795, Japan

**Keywords:** adaptation, alkaloid, biotic stressor, drought, flooding, photosynthesis, reproduction, salinity, stress condition

## Abstract

*Arundo donax* L. has been introduced in markets worldwide due to its economic value. However, it is listed in the world’s 100 worst alien invasive species because it easily escapes from cultivation, and forms dense monospecific stands in riparian areas, agricultural areas, and grassland areas along roadsides, including in protected areas. This species grows rapidly and produces large amounts of biomass due to its high photosynthetic ability. It spreads asexually through ramets, in addition to stem and rhizome fragments. Wildfires, flooding, and human activity promote its distribution and domination. It can adapt to various habitats and tolerate various adverse environmental conditions, such as cold temperatures, drought, flooding, and high salinity. *A. donax* exhibits defense mechanisms against biotic stressors, including herbivores and pathogens. It produces indole alkaloids, such as bufotenidine and gramine, as well as other alkaloids that are toxic to herbivorous mammals, insects, parasitic nematodes, and pathogenic fungi and oomycetes. *A. donax* accumulates high concentrations of phytoliths, which also protect against pathogen infection and herbivory. Only a few herbivores and pathogens have been reported to significantly damage *A. donax* growth and populations. Additionally, *A. donax* exhibits allelopathic activity against competing plant species, though the allelochemicals involved have yet to be identified. These characteristics may contribute to its infestation, survival, and population expansion in new habitats as an invasive plant species. Dense monospecific stands of *A. donax* alter ecosystem structures and functions. These stands impact abiotic processes in ecosystems by reducing water availability, and increasing the risk of erosion, flooding, and intense fires. The stands also negatively affect biotic processes by reducing plant diversity and richness, as well as the fitness of habitats for invertebrates and vertebrates. Eradicating *A. donax* from a habitat requires an ongoing, long-term integrated management approach based on an understanding of its invasive mechanisms. Human activity has also contributed to the spread of *A. donax* populations. There is an urgent need to address its invasive traits. This is the first review focusing on the invasive mechanisms of this plant in terms of adaptation to abiotic and biotic stressors, particularly physiological adaptation.

## 1. Introduction

**Figure 1:**
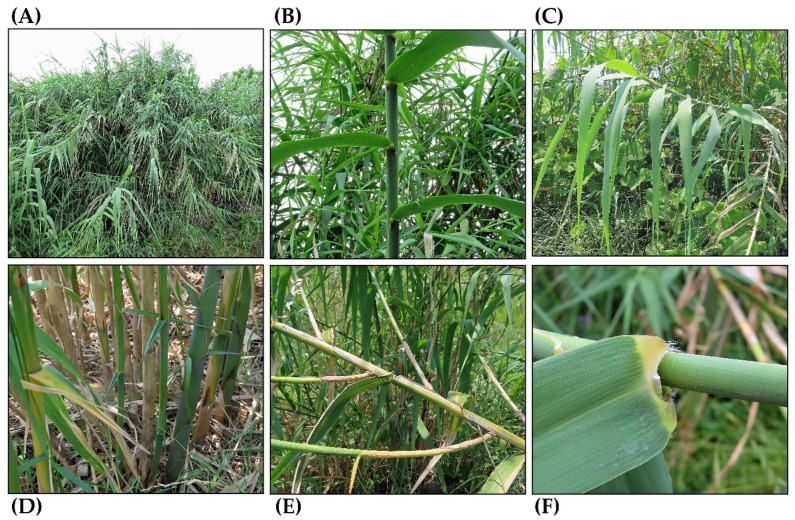
*Arundo donax.* (**A**) Stand; (**B**) base of the stand; (**C**) stem; (**D**) branches (**E**) leaves; (**F**) leaf base and ligule.

*Arundo donax* L., belonging to the Poaceae family, is a perennial cane grass commonly known as a giant reed, giant cane, Spanish cane, bamboo reed, and many other local names. Its stem (culm) grows to a height of 3–9 m, and its hollow internodes are 2–20 cm long and 1.5–3.5 cm in diameter. The stems become woody and develop branches from axillary buds at the nodes with maturity. The leaves are distichous phyllotaxis, measuring 30–80 cm in length and 2–12 cm in width. They have glabrous sheaths that split to the leaf bases, and membranous leaf ligules. The sheaths tightly envelop the stem from the base of the leaf to the next lower node [1,2,3,4,5,6]. The membranous leaf ligules are one of the features that distinguish this species apart from similar *Phragmites* species [5]. This species has well-developed rhizomes. The rhizomes are horizontal, 3.5–5 cm thick, and solid, forming a dense network. Each stem has a lifespan of three to four years. However, the rhizomes survive and continuously produce ramets that grow into new shoots with leaves and stems [5,6,7] (Figure 1).

Since ancient times, *A. donax* has been cultivated throughout the Mediterranean Basin, the Middle East, and South and East Asia. It has been used to make woodwind reeds, furniture, and household items, such as baskets, containers, and mats. It has also been used for construction materials and agricultural structures, including for those that support orchards and crops [5,7]. Genetic and morphological analyses have identified *A. donax* as an invasive archaeophyte in the Mediterranean Basin. Populations of *A. donax* in the Middle East and in South and East Asia exhibit significant genetic and morphological variation. In contrast, populations of *A. donax* in the Mediterranean Basin exhibit lower genetic and morphological variation [5,7,8,9,10]. The chromosome number of *A. donax* has been reported to be approximately 108 (2*n* = 18*x*) in the populations from the Mediterranean Basin, and 72 (2*n* = 12*x*) in the populations from the Middle East [11,12,13]. Defective development of the female and male gametophytes has been observed in the populations from the Mediterranean Basin, resulting in sterility [12,14,15]. Therefore, *A. donax* is thought to be native to the Middle East and South and East Asia. The ploidy level of *A. donax* has also been reported as 2*n* = 7*x* = 84 in Australian populations [16], and 3*n* = 9*x* = 108 (autoalloenneaploid) in Chinese populations [17,18]. Further global-scale investigations into the chromosomes of this species may be imperative.

European colonialists widely introduced *A. donax* from the Mediterranean Basin to tropical, subtropical, and warm temperate areas around the world [2,5,7]. It was introduced to North America in the early 1500s for textile production. By the early 1800s, it had spread to California for applications in erosion control and windbreaks. It was introduced to South Africa for applications in erosion control in the 1700s, and to Australia in the 1800s [2,19,20,21]. In addition to its economic benefits, *A. donax* has ornamental value, and the potential to produce bioenergy [22,23,24,25]. It has already been distributed over 100 countries in the tropical, subtropical, and warm temperate regions of Europe, North America, South America, Africa, and Oceania [2,5,7,26].

**Figure 2:**
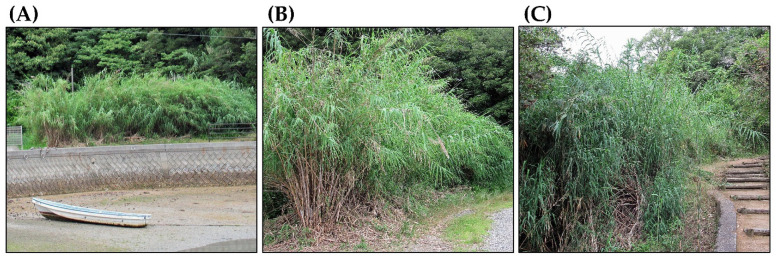
*Arundo donax* habitats. (**A**) Bay area; (**B**) roadside; (**C**) hillside.

However, *A. donax* easily escapes from cultivation during high-water events, such as flooding. It also escapes through human activities. It forms dense monospecific stands in riparian habitats, including floodplains, streams, and channels. It also grows in agricultural areas and along roadsides and hillsides, including protected areas [2,5,7,21]. In Europe, *A. donax* populations are found in countries ranging from the Mediterranean region to Germany [22]. In North America, populations are distributed across 39 U.S. states, ranging from California to New York, and from Texas to northern Portland, as well as in Hawaii. They are also found across 27 states in Mexico [27,28]. Infestations are estimated to cover 6400 and 8400 hectares in the Rio Grande Basin in Texas and in Mexico, respectively, and 4100 hectares in California [29,30,31]. Currently, *A. donax* has been found along the entire river channels, from bank to bank, in the coastal river basins in Southern California [32]. *A. donax* is considered to be one of the worst invasive plants because of the risks it poses to native flora and fauna. It is listed among the world’s 100 worst invasive alien species [33] (Figure 2).

Management strategies for *A. donax* have been reviewed and summarized in detail [5,7]. Control measures include chemical, physical, mechanical, biological, and ecological methods. Eradicating the species from these infested areas seems difficult, and relying solely on the existing methods is unsafe and unsustainable at the ecosystem level [7,34,35]. Restoring the flora and fauna, which negatively impacted by *A. donax*, is also difficult [36,37]. Long-term, continuous integrated management programs utilizing new technology are necessary to eradicate *A. donax*. Understanding the biology of the species may be a key to controlling it [38,39]. The life history traits of several invasive plant species have been studied to understand their invasion mechanisms. These mechanisms include growth and reproductive capabilities, as well as their adaptive abilities in response to abiotic and biotic stressors [40,41,42,43,44,45,46,47]. Understanding the invasion mechanisms of *A. donax* is also important for the long-term integrated weed management programs.

This review provides an overview of the growth and reproductive capacity of *A. donax*, and its adaptability to abiotic and biotic stressors. It also discusses the impact of *A. donax* on abiotic and biotic ecosystem processes.

## 2. Literature Survey

A combination of major online search engines was used to review the literature: Scopus, ScienceDirect, and Google Scholar. The following terms were searched in combination with *Arundo donax*: invasion, ecology, distribution, impact, genetic variation, growth, reproduction, habitat, adaptation, flooding, temperature, drought, salinity, herbivory, pathogens, allelopathy, toxicity, and alkaloids. We discuss the identified papers as thoroughly as possible. We excluded those with unclear methods.

## 3. Life History Traits of *Arundo donax*

### 3.1. Growth

*A. donax* is known for its rapid growth. It can grow 2.6–10 cm per day, making it one of the fastest-growing terrestrial plants [48,49]. *A. donax* is a C_3_ plant with a photosynthetic CO_2_ assimilation rate of 33–38 μmol m^−2^ s^−1^ under optimal conditions [50,51,52,53]. This rate exceeds the standard value of 18–20 μmol m^−2^ s^−1^ for C_3_ plants [54]. The high photosynthetic ability of *A. donax* may be due to its ability to absorb high levels of irradiance for the electron transport saturation, and its high levels for ribulose-1,5-bisphosphate (RuBP)-limited and RuBP-saturated photosynthesis [55,56].

It has been estimated that *A. donax* produces 125 tons of above-ground biomass (dry weight) per hectare in one growing season in Australia [57]; 23–172 tons of above-ground biomass in Western and Southeastern U.S. [58,59,60,61]; 36–167 tons of above-ground biomass in India [62]; 22–137 tons of above-ground biomass in the Nile Delta in Egypt [63]. Estimates of European populations range from 15 to 63 tons of above-ground biomass in Spain [64,65]; 5 to 51 tons in Italy [66,67,68]; 20 to 25 tons in France [22]; 7 to 26 tons in Germany [22]; 7 to 22 tons in Greece [69]; 13 to 53 tons in Turkey [70,71]. Production in European populations was lower than in other areas. Different levels of solar irradiation and temperature may be important factors in determining total above-ground biomass [72,73]. Therefore, *A. donax* grows rapidly and produces large amounts of biomass. One reason for this is its high photosynthetic ability.

### 3.2. Reproduction and Establishment

**Figure 3:**
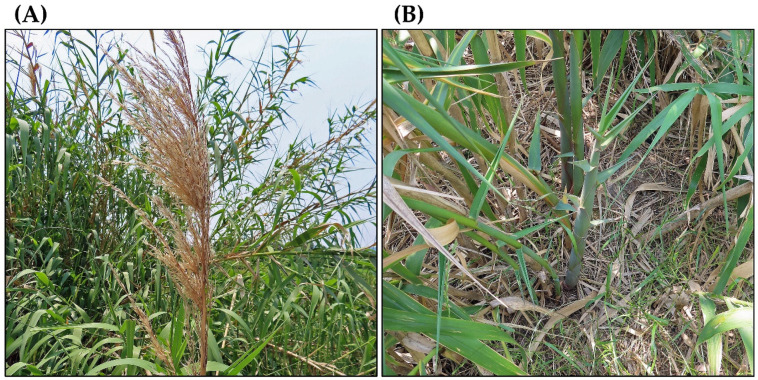
*Arundo donax.* (**A**) Panicle; (**B**) ramet.

The flowering of *A. donax* is either regular or irregular, depending on the environment and population. The panicles, 30–80 cm long and 5–30 cm wide, are generated at the end of the stems (culms), and contain a large number of spikelets. The spikelets are 10–15 mm long and have two to four bisexual florets. However, the populations in the Mediterranean region, USA, Australia, and India have not been observed to produce viable seeds [12,16,74,75,76,77]. Defective development of the female and male gametophytes, including failure of the meiotic processes of these gametophytes, has been observed in these populations, causing sterility [12,14,78]. Viable seeds of *A. donax* have been reported in Pakistan, Afghanistan, and Iran [79], and the germination of seeds from Chinese populations has also been observed [80,81]. However, detailed information on the production and germination of viable seeds of *A. donax* in its native ranges is limited. According to the genetic and ecological analyses, some *A. donax* populations in central Mexico were implied to reproduce sexually [82]. The seed production and germination processes of *A. donax* still need to be investigated globally in the future (Figure 3).

*A. donax* spreads asexually through ramet sprouting [5,7,31]. New ramets arise from adventitious buds at the nodes of rhizomes (Figure 3). In California, individual *A. donax* plants were observed to expand by 0.3–0.5 m per year via rhizome ramets. *A. donax* populations expanded by 4.7 hectares per year through rhizome ramets [83]. When *A. donax* stems come into contact with the soil due to some environmental conditions, such as flooding or strong winds, ramets and nodal roots emerge from the adventitious buds at stem nodes. Independent clonal plants form when the rooted nodes detach from the mother plants due to the death of the layering stems. Expansion by layering stems was 7.4 times faster than rhizome expansion [83]. These vegetative reproductions may contribute to the horizontal expansion and the formation of the dense monospecific stands.

Stem and rhizome fragmentation of *A. donax* is often observed following flooding and other disturbance to its stands [2,31]. If the fragments contain at least one node, they can easily sprout ramets and establish new stands. However, the ability to sprout is affected by temperature, moisture, and the time since fragmentation [84,85,86]. Reproduction through fragmentation contributes to the long-distance expansion of *A. donax* populations. Following significant flooding, the populations expanded into coastal river basins in Southern California [31,87]. A molecular analysis of *A. donax* populations along the Santa Ana River in California suggested that most of these populations were spread along the river via vegetative reproduction [88]. Similarly, *A. donax* populations in Southern Australia spread along rivers via vegetative reproduction [16]. Rhizome fragments establish *A. donax* populations much more frequently than stem fragments [89]. Following flooding, 57% of the vegetative reproduction along the Santa Margarita River originated from rhizome fragments. Of that, 33% came from stem fragments. The remaining 7% could not be identified [90]. Populations of *A. donax* have also increased due to stem and rhizome fragmentation caused by human activity, such as the use of bulldozers and other equipment. It is also an escaped garden plant [91,92]. Dumping garden waste along roads, canals, and field borders is a recognized method by which *A. donax* spreads [91,92]. Most of the invasive populations in Southern California have been identified as the result of plants escaping or being displaced from commercial plantations and horticultural propagation. These plants then spread along rivers and roads via vegetative propagules [31].

The reestablishment of *A. donax* after a wildfire was observed on 300 hectares of riparian forest along the Santa Clara River in Southern California. The plant sprouted from its rhizomes shortly after the fire, and reached heights of up to 2.3 m within three months. In contrast, the emergence of native woody riparian plants was not observed for two months after the fire. One year later, the density of the *A. donax* population was 20 times higher than that of the native woody plants [48]. Wildfires have also been observed to promote the spread of *A. donax* into native riparian habitats in the Central Valley and coastal watersheds of California [93,94]. Therefore, wildfires promote *A. donax* infestation by replacing native vegetation with *A. donax*-dominant vegetation.

*A. donax* expands its stands horizontally, and forms dense monospecific stands through the ramets from the adventitious buds of its rhizomes, and layering stems. Reproduction through stem and rhizome fragments, which are generated by flooding and human activity, contributes to the long-distance expansion of *A. donax* populations. Wildfires also promote the spread of *A. donax* into native vegetation and its domination.

## 4. Adaptation of *Arundo donax* to Abiotic Stress

*A. donax* adapts to tropical, subtropical, and warm temperate climates. It grows in gravelly soils, coarse sands, clays, and river sediments. It inhabits areas ranging from well-drained soils to moist soils, including those where the water table is near the surface. It also grows in areas with freshwater–semi-saline water. The pH of these soils ranges from 5.0 to 8.7 [2,95]. *A. donax* maintains high biomass production under conditions of low soil nutrient availability [96]. The plant grows in coastal river basins, thickets, estuaries, and on sand dunes near seashores. It grows along riverbanks, in lakes, on floodplains, in agricultural areas, in irrigation canals, along field borders, in grasslands, along roadsides and hillsides, in open forests, and in other moist disturbed areas [2,91,97] (Figure 2). It is cultivated as an ornamental and industrial crop in gardens and fields [31,98]. Therefore, *A. donax* grows in a wide range of habitats. It is also tolerant of various abiotic stresses such as cold temperatures, drought, flooding, and salinity.

### 4.1. Cold Temperature

*A. donax* thrives in temperatures ranging from approximately 10 to 30 °C. It does not grow well in areas with winter frost. Its above-ground parts die in cold temperatures. However, *A. donax* can survive cold temperatures by maintaining its underground parts such as rhizomes and roots. Is sprouts ramets from the axillary buds at the rhizome nodes when the temperature becomes adequate [2,22,31,91,97]. It has been reported that *A. donax* can survive at temperatures as low as −10 °C if it is acclimated to 10 °C for at least one week. During this time, its total soluble sugar concentration increases, giving it tolerance [99]. The global warming trend may further expand the *A. donax* populations northward in the Northern Hemisphere and southward in the Southern Hemisphere [100].

### 4.2. Drought

*A. donax* grows in areas ranging from highly humid to dry, with long drought periods, and with annual precipitation ranging from 300 to 4000 mm [2,101]. The species adapts to the drought stress conditions through morphological and physiological changes. Under these conditions, *A. donax* produces small thick leaves, but does not reduce its leaf number [102,103]. Smaller leaves have lower water consumption and transpiration rates. This results in higher water-use efficiency than that of larger leaves [104,105,106]. Water-use efficiency is the ratio of total biomass production to total water consumption. Under conditions of 20% available water, *A. donax* increased the water-use efficiency index by 1.5-fold compared to the control treatment [103]. Under drought stress conditions, *A. donax* also reduces above-ground biomass rather than below-ground biomass for its survival [102,103]. Some *A. donax* ecotypes have reduced xylem vessel size and impaired water transport, enabling the plants to grow under moderate drought conditions [106].

Under drought stress conditions, *A. donax* reduces the osmotic potential of its leaves; however, their water content does not differ from that of control leaves [103]. This suggests that the lower osmotic potential was due to the osmotic adjustment through solute accumulation [107,108]. Osmotic adjustment contributes to the maintenance of turgor in cells and plant vigor under drought conditions [109].

Drought stress increases the production of in the different cellular compartments, such as chloroplasts, mitochondria, and peroxisomes. This results in extensive cellular damage and death. Since solar light energy is received and transferred to light-harvesting systems (photosystems I and II) on the chloroplast thylakoid membranes, chloroplasts are the main cellular sites for ROS production [110]. Under drought conditions, *A. donax* reduces ROS production by effectively controlling stomatal conductance through an increase in the content of the plant hormone ABA in the leaves [111,112]. Drought stress was found to not seriously damage the photosynthetic apparatus of *A. donax*, allowing it to maintain photosynthesis [103]. These morphological and physiological changes in *A. donax* may be adaptations that increase water-use efficiency and survival under drought stress conditions.

### 4.3. Flooding

*A. donax* often suffers from waterlogging conditions in the riparian habitats during high-water events, including flooding. This results in a low oxygen condition (hypoxia) for the roots and rhizomes of *A. donax* [113]. Under stress conditions, *A. donax* switches from aerobic respiration to alternate anaerobic respiration [113,114]. This process produces supplemental energy [115,116,117,118] and enables *A. donax* to grow [119]. Intensive oxygen deprivation reduces the growth and photosynthesis of *A. donax.* However, the re-oxygenation rapidly restores its physiological functions [119]. Prolonged waterlogging has also been shown to induce aerenchyma development in the roots of *A. donax* [113,120]. Aerenchyma forms in the roots and shoots of wetland species under low-oxygen conditions. Root aerenchyma delivers oxygen to the root tips and contributes to their survival under these conditions [121,122]. These physiological and morphological adaptations to waterlogging stress likely explain why monospecific *A. donax* stands are so widespread in wetlands, which frequently experience waterlogging conditions.

### 4.4. Salinity

Salinity stress negatively affects plant growth and photosynthesis. It can even cause death [123,124,125]. *A. donax* exhibited decreases its growth and biomass production as salinity increases; however, the plants show high tolerance to salinity stress conditions [102,126,127]. At a salinity level of 12 dS m^−1^, *A. donax* growth decreased by 50% compared to the control. Photosynthetic CO_2_ assimilation rates were maintained at 7–12 μmol m^−2^ s^−1^ under salinity levels of 38–42 dS m^−1^. These rates are 18–36% of those under optimal conditions (33–38 μmol m^−2^ s^−1^) [50,53,102,111,126]. Its mortality rate was not recorded, even at high salinity levels [128,129,130,131]. Soils are classified as saline when their electrical conductivity exceeds 4 dS m^−1^, which is equivalent to 40 mM of sodium chloride, generating an osmotic pressure of approximately 0.2 MPa. The electrical conductivity of seawater is 20–50 dS m^−1^ [132]. Therefore, *A. donax* exhibited relatively high growth and photosynthetic capacity under high-salinity conditions.

Salinity stress is primarily caused by the osmotic effect of the salt around plant roots. This results in the temporary loss of water in leaf cells, producing effects similar to those of drought stress [123,124,125]. In *A. donax,* salinity stress induces stomatal closure to decrease transpiration by increasing ABA synthesis. Stress also increases sucrose production and gene expression related to carbohydrate metabolism in *A. donax* leaves [133]. Sucrose may counteract the increase in osmotic potential caused by salinity stress, thereby decreasing it. Stress also increases zeaxanthin content in the leaves [133]. Zeaxanthin is a carotenoid and yellow pigment that acts as a light energy modulator and a photochemical quenching molecule [134,135]. Thus, zeaxanthin protects *A. donax* against the oxidative stress caused by salinity, including ROS production, as described in Section 4.2. *A. donax* is well adapted to coastal riparian environments, where salinity levels are often high due to tidal waves. Consequently, some authors have categorized *A. donax* as a halophyte due to its salinity tolerance [136,137,138].

## 5. Adaptation of *Arundo donax* to Biotic Stresses

Biotic stressors affect the germination, growth, development, and reproduction of plants. These stressors include herbivores, pathogens, and competing plant species. These stressors exert significant selective pressure on plant survival, abundance, and distribution [139,140,141,142,143,144]. Several invasive plant species have been documented to exhibit defense responses, and these defensive mechanisms likely contribute to increasing their populations and distribution in the introduced range [145,146,147,148]. *A. donax* has also been reported to exhibit toxic activity against various biotic stressors.

### 5.1. Defensive Response Against Herbivores and Pathogens

**Figure 4:**
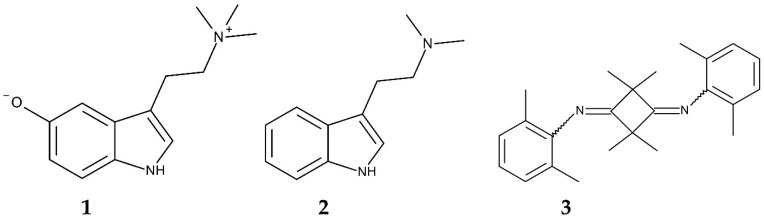
Compounds involved in the defense functions of *Arundo donax.* **1**: Bufotenidine; **2**: gramine; **3**: 2,2,4,4-Tetramethyl-*N,N*-bis(2,6-dimethyl-phenyl)-cyclobutane-1,3-diimine.

The first reports of *A. donax* poisoning occurred in rats (*Rattus norvegicus*) and mice (*Mus musculus*). The main active component responsible for the toxicity was identified as bufotenidine, an indole alkaloid. Administrating 10 mg kg^−1^ of bufotenidine caused the sudden death in mice and the ataxia in rats [149]. Bufotenidine acts as a selective serotonin 5-HT_3_ receptor blocker. These receptors are involved in a wide range of functions, including those of the central nervous system. Blocking these receptors causes various adverse physiological and behavioral changes in mammals [150,151,152,153] (Figure 4). However, the distribution and concentration of bufotenidine in *A. donax* remains unknown.

As another indole alkaloid, gramine has been identified in the leaves, flowers, and rhizomes of *A. donax* [149,154,155] (Figure 4). A dosage of over 0.3 g kg^−1^ of gramine was found to decrease the feed intake and body weight gain of rats [156]. Gramine exhibits insecticidal activity against various herbivorous insects, including aphids (*Rhopalosiphum padi* and *Sitobion avenae*), the European elm bark beetle (*Scolytus multistriatus*), the brown planthopper (*Nilaparvata lugens*), and the cotton bollworm (*Helicoverpa armigera*) [157,158,159,160]. Gramine has been demonstrated to have antifungal activity against the pathogen *Fusarium graminearum* [161]. Additionally, the extracts of *A. donax* and gramine induced reactive oxygen species (ROS) and malondialdehyde and catalase activity, while decreasing superoxide dismutase in the freshwater cyanobacterium *Microcystis aeruginosa* [162,163]. These results suggest that the extracts and gramine induce oxidative stress in *Microcystis aeruginosa. M. aeruginosa* causes toxic blooms [164,165]. The concentration of gramine in the shoots of *A. donax* was found to be 17.3 nmol g^−1^ of fresh *A. donax* weight [166]. However, the mechanism by which gramine exerts its toxic effects remains unclear.

Several other alkaloids were found in *A. donax* [167,168]. One of these alkaloids, 2,2,4,4-tetramethyl-*N,N*-bis(2,6-dimethyl-phenyl)-cyclobutane-1,3-diimine, exhibited antifeedant activity against the boll weevil *Anthonomus grandis* [169,170] (Figure 4). Extracts of *A. donax* increased the mortality of caddisfly larvae, *Lepidostoma unicolor* [171], and the intestinal parasitic nematode *Haemonchus contortus* [172]. The extracts also inhibited the growth of the oomycete pathogen *Pythium ultimum* [173]. *Pythium ultimum* infests a wide range of the host plants, causing root rot and damping-off diseases [174,175]. However, the active components responsible for these toxic effects have not yet been identified.

In addition, *A. donax* accumulates high concentrations of phytoliths, which are microscopic amorphous silica particles, in its leaves and stems [176,177]. Plant roots absorb silicon (Si) as monosilicic acid (H_4_SiO_4_) from groundwater. The vascular system then carries the monosilicic acids to different parts of the plant, where phytoliths form through a process called biosilicification [178,179,180]. Phytoliths in the plant tissues play a defensive role against fungal pathogen infections and herbivory by insects and mammals [181,182]. Thus, the phytoliths contribute to the survival of *A. donax* from pathogen infection and herbivory.

Furthermore, only a limited number of herbivores and pathogens affecting *A. donax* have been reported, and the specific damage they cause is unclear [2,31,183]. Moderate–low levels of leaf damage and defoliation alone do not significantly impact the survival or growth of *A. donax.* These levels of damage may not reduce the *A. donax* population in a given habitat [184]. Therefore, herbivores and pathogens may not significantly impact the growth and population of *A. donax.* Toxic substances, including bufotenidine, gramine, other alkaloids, and phytoliths may contribute to the plant’s defenses against herbivores and pathogens.

### 5.2. Defensive Response Against Competing Plant Species

Many invasive plant species have been reported to inhibit the germination and growth of competing plants through allelopathy, which involves the release of allelochemicals [185,186,187,188,189]. This suppression of neighboring plants gives these invasive plants an advantage in acquiring resources, such as light, water, and nutrients. These plants synthesize and store allelochemicals in various parts of the plant, including the leaves, stems, and rhizomes. They release these allelochemicals into neighboring environments as necessary [190,191,192,193,194,195,196]. The allelopathic activity of *A. donax* was determined using its extracts. Aqueous extracts of *A. donax* leaves suppressed the germination and growth of *Lens culinaris* under laboratory and greenhouse conditions [197]. Aqueous extracts of *A. donax* leaves and rhizomes suppressed the growth of the grass species *Megathyrsus maximus*, and five woody species, *Pseudobombax tomentosum* (Malvaceae), *Guazuma ulmifolia* (Sterculiaceae), and *Parkia platycephala* (Malvaceae), *Eriotheca pubescens* (Malvaceae), and *Handroanthus impetiginosus* (Bignoniaceae). The inhibitory activity of the leaf extracts was greater than that of the rhizome extracts [198]. However, the allelochemicals responsible for this activity have not yet been identified.

Additionally, methanol extracts from the above-ground parts of *A. donax* inhibited the growth of the haptophyte *Prymnesium parvum,* and gramine was identified as a toxic substance in the extracts [199]. *P. parvum* causes toxic algal blooms [200]. Gramine inhibited the growth of *P. parvum* with 50% growth inhibition values of 2.78 μg mL^−1^. Gramine also suppressed the growth of the raphidophyte *Chattonella marina,* which is associated with red tides [201], as well as the green alga *Desmodesmus armatus* [202].

Based on these observations, *A. donax* appears to exhibit allelopathic activity. Identifying the allelochemicals of *A. donax* will be necessary in the future to understand its defensive response against competing plant species. *A. donax* is also toxic to certain alga species, including haptophytes, raphidophytes, and green algae. Gramine also exhibited toxicity toward these algae. However, the ecological significance of this toxicity remains unclear.

## 6. Impacts of *Arundo donax* on Abiotic and Biotic Ecosystem Processes

*A. donax* forms dense monospecific stands in riparian areas, such as floodplains, riverbanks, streams, and channels, including those in protected areas. This alters the structure and function of these ecosystems, including their abiotic and biotic components [2,5].

### 6.1. Impacts on Abiotic Ecosystem Processes

The estimated water consumption was 9.1 mm per day for the *A. donax* populations in the Rio Grande Basin in Texas, and 41.1 mm per day for the populations in Southern California [31,203]. Along the Nueces River in Texas, the water uptake of *A. donax* populations was 7.2% higher than that of the native grass species *Panicum virgatum,* which was displaced by *A. donax* infestation [204]. The *A. donax* population (405 hectares) along the Santa Margarita River in California consumes 1100 mm of water per year, which is equivalent to the annual consumption of a downstream community of 20,000 inhabitants [19]. Therefore, *A. donax* infestations reduce river flow and the amount of available water for residents, agriculture, and industry.

The stem density of *A. donax* has been reported to range from 17 to 400 stems per m^2^, with an average of 130 stems per m^2^ in California watersheds [205], and 63 stems per m^2^ along the Russian River in California [206]. The dense stands of *A. donax* within streams affect water movement, disturb irrigation systems, and increase stream sedimentation. This narrows the channels and increases flood risk [207,208].

The woody plant species *Salix laevigata* used to dominate the riverbanks along the lower Santa Clara River in California. However, an *A. donax* infestation occurred. The two species have significantly different belowground architectures. *S. laevigata* has much stronger and deeper roots than *A. donax.* The root tensile strength of *S. laevigata* is five times higher. Banks with *S. laevigata* are more than twice as stable as banks with *A. donax.* Therefore, *A. donax* weakens the stability of the river and channel banks and increases erosion during high-flow events [207]. Additionally, *A. donax* collapses into rivers and channels during these events. Large masses of debris plug bridges and form sandbars and dams. This prevents flood control structures from functioning properly and increases flood risk [205,207,209].

Due to its lower moisture content, higher surface-to-volume ratio, and dense biomass, including senescent and dead stems, *A. donax* increases fuel load and fire risk and intensity [19,210,211]. The frequency of wildfires is positively correlated with the areas occupied by *A. donax* [31,61]. It is also believed that *A. donax* modifies the microclimate of its infested areas by raising temperatures and reducing dissolved oxygen levels in the water. Unlike native forests, *A. donax* stands do not form a canopy. This allows direct solar irradiation of the water surface, and affects the climate [5,19]. However, detailed climate information, including solar irradiation in the *A. donax*-infested areas, is unavailable.

As described herein, *A. donax* has negative impacts on the hydrology and geographical features of infested areas. These effects include reduced water availability, increased erosion and flooding, and increased fire risk and intensity in riparian areas.

### 6.2. Impacts on Biotic Ecosystem Processes

*A. donax* outcompetes native plant species and forms its dominant vegetation. Dense monospecific stands of *A. donax* threaten the native flora in infested habitats. Compared to uninfested areas, *A. donax* significantly reduces plant diversity [31,212,213,214,215,216]. The presence of dense *A. donax* stands also results in a significant lack of understory vegetation. In coastal Southern California watersheds, *A. donax* has been observed displacing all vegetation, including shrubs and herbs except mature tall trees [31]. *A. donax* has replaced *Salix laevigata* forests along the lower Santa Clara River in California [208], and *Panicum virgatum* grasslands along the Nueces River in Texas [204]. In California, *A. donax* endangers protected plant species in riparian areas, such as *Ambrosia pumila, Arenaria paludicola, Atriplex coronata* var. *notatior,* and *Berberis nevinii* [31]. As described in Section 6.1, fire and flood events are exacerbated by *A. donax* infestation. Following these events, the removal of riparian vegetation leads to the dominance of *A. donax* and alters plant succession patterns [94]. The *A. donax* population also expands through stem and rhizome fragments along riverbanks after flooding as described in Section 3.2. These positive feedback loops may contribute to the dominance of *A. donax*.

Replacing native vegetation with *A. donax* also alters the structure and function of ecosystems. Dense monospecific stands of *A. donax* negatively impact fauna by affecting the food availability, nesting opportunities, movement, and the food chain and trophic level modifications. *A. donax* infestation endangers several protected amphibians, fish, birds, mammals, and other invertebrates and vertebrates [31,216].

The abundance and diversity of ants and beetles were much lower in *A. donax*-dominant vegetation than in native vegetation along the Rio Grande Basin in Texas [217,218]. The abundance and taxonomic richness of aerial invertebrates, as well as that measured with the Shannon Index (which indicates species richness and evenness within a community), were twice as high in the native riparian vegetation as in the *A. donax*-dominant vegetation at Sonoma Creek in California. Insects recorded in *A. donax* vegetation rarely exhibit feeding activity [219]. The abundance and species composition of arthropods in *A. donax*-dominant vegetation was significantly lower than in native vegetation in riparian areas across Mediterranean rivers in Spain due to poor understory vegetation and litter accumulation. Some entire trophic groups have even been lost in *A. donax*-dominated vegetation areas [220]. The greater species diversity and abundance of arthropods in native riparian vegetation compared to *A. donax*-dominant vegetation may be due to the greater habitat structure, including thick litter layers, and the higher-quality food availability. *A. donax* produces a large amount of biomass. However, it provides limited food availability and a short food chain, which significantly impacts invertebrate populations. Additionally, the number of non-native invertebrate species associated with *A. donax* infestation increased [221].

The presence of *A. donax* creates physical barriers that disturb the movement of amphibians between the riparian habitats and upland habitats. Since amphibians transform from aquatic larvae into air-breathing adults; so, migration from aquatic to upland environments is necessary. *A. donax* endangers protected species of amphibians, such as the arroyo toad (*Bufo californicus*) and the mountain yellow-legged frog (*Rana muscosa*) in the riparian areas in California [31,222].

*A. donax* endangers fish species, such as the rainbow trout (*Oncorhynchus mykiss*) and the northern tidewater goby (*Eucyclogobius newberryi*) in California riparian areas [31]. As discussed in Section 6.1, *A. donax* affects water movement in terms of hydrology and geomorphology. As mentioned in Section 5.2, the plant produces toxic substances that can contaminate water through the residue. These alterations to hydrology and geomorphology, as well as the presence of these toxic substances, may affect fish populations.

The abundance and species richness of birds in California riparian areas decreased as *A. donax* increased [223]. This invasive species endangers several protected bird species, including the least Bell’s vireo (*Vireo bellii pusillus*) and the southwestern willow flycatcher (*Empidonax traillii extimus*) [31,216]. Nesting opportunities for the least Bell’s vireo in *A. donax* stands were less than 1% of those in native shrub and tree vegetation, such as *Salix laevigata* and *Baccharis salicifolia*. The physical structure of *A. donax,* such as its branching, differs greatly from that of native shrubs and trees [31]. The impact of *A. donax* on birds may be due to its physical structure, and the reduction in available invertebrates for food. The detection of carnivores, such as coyotes (*Canis latrans*) and bobcats (*Lynx rufus*), is significantly lower in *A. donax* dominated habitats than in native habitats in the Santa Clara River Valley in California, due to limited movement and hunting opportunities for small animals [224]. *A. donax* has complicated effects on herbivorous mammals. While *A. donax* stands may provide hiding places from carnivores, food resources are lower compared to native plants due to a lack of seeds and low-quality forage [224].

An *A. donax* infestation significantly reduces plant diversity and richness. It also alters the structure and function of ecosystems, decreasing the fitness of habitats for invertebrates and vertebrates, including arthropods, amphibians, fish, birds, and mammals. This decrease in fitness leads to a reduction in species diversity and richness. However, the effects of *A. donax* infestation on soil microorganisms remain unclear. These microorganisms may play an important role in ecosystem functions.

## 7. Conclusions

*A. donax* has been introduced to over 100 countries in the tropical, subtropical, and warm temperate regions across Europe, North America, South America, Africa, and Oceania [2,5,7,26]. One reason for its introduction is its economic value [5,7]. However, it easily escapes from cultivation, and forms dense monospecific stands in riparian areas, agricultural areas, and roadsides, including protected areas [2,5,7,31]. *A. donax* is listed among the world’s 100 worst invasive alien species due to the risks it poses to native flora and fauna [33]. *A. donax* grows rapidly and produces large amounts of biomass due to its high photosynthetic ability. It is considered to be one of the fastest-growing terrestrial plants [48,49,50,51,52,53]. This species spreads asexually through the ramets from adventitious buds at the rhizome and stem nodes, forming dense stands [31,83]. Stem and rhizome fragments can easily sprout ramets, establishing new plants and populations along rivers, and promoting long-distance distribution [16,31,87,88,89,90]. Wildfires, flooding and human activity promote the distribution and domination of *A. donax* [16,48,88,92]. However, information on the production and germination of viable *A. donax* seeds in its native range is limited. Further research is needed to investigate *A. donax* seeds production and germination globally.

*A. donax* can adapt to the various habitats and tolerate to various adverse environmental conditions [2,91,95,96,97,98]. It thrives in temperatures ranging from 10 to 30 °C. However, it can survive in cold temperatures by increasing its sugar concentration and sprouting ramets, even after dieback [2,22,31,91,97,99]. The species tolerates drought stress through morphological and physiological adaptations. It produces small, thick leaves to maintain water-use efficiency. It also maintains vigor and photosynthesis by adjusting osmotic potential and avoiding ROS production [102,103,105,108,111,112]. Under flooding conditions, *A. donax* switches from aerobic to anaerobic respiration, and produces supplemental energy. Re-oxygenation rapidly enhances its physiological functions [113,114,115]. The species also induces aerenchyma during prolonged waterlogging conditions [113,120]. Aerenchyma contributes to oxygen movement [121,122]. *A. donax* also has strong salinity tolerance due to physiological adaptations that increase gene expression related to salinity tolerance, maintaining its osmotic potential [126,127,128,129,130,131,133]. Because of its high salinity tolerance, the species is categorized as a halophyte [136,137,138].

*A. donax* exhibits defense responses against biotic stressors, including herbivores, pathogens, and competing plant species. This species produces indole alkaloids, such as bufotenidine and gramine, as well as the alkaloid, 2,2,4,4-tetramethyl-*N,N*-bis(2,6-dimethyl-phenyl)-cyclobutane-1,3-diimine [149,154,155,169]. These alkaloids are toxic to herbivorous mammals, insects, parasitic nematodes, and pathogenic fungi and oomycetes [149,156,157,158,159,160,161,162,163,164,165,169,170,171,172,173,174,175]. *A. donax* also accumulates high concentrations of phytoliths [176,177], which protect against pathogen infection and herbivory. Only a few numbers of herbivores and pathogens have been reported to cause significant damage to the growth and populations of *A. donax* [2,31,183]. Therefore, *A. donax* may have strong defense mechanisms against biotic stressors. It also exhibits allelopathic activity against competing plant species [197,198] (Table 1).

The characteristics of *A. donax*, such as its rapid growth and reproductive ability, adaptability to various stress conditions, and defense functions against biotic stressors, may contribute to its infestation, survival, and population expansion in new habitats as an invasive plant species. These characteristics make *A. donax* difficult to control with a single control measure [5,7,31,34,35,36,37,38,39]. Eradication of *A. donax* from a habitat requires an ongoing long-term integrated management approach based on its invasive mechanisms. This approach should incorporate smart decision-making tools, innovative equipment, various herbicides, and other methods [225,226,227,228,229,230]. This is the first review to focus on the invasive mechanisms in terms of adaptation to abiotic and biotic stressors, particularly physiological adaptation. However, the mechanisms of mode of action of toxic substances against pathogens and herbivores are unclear. The allelochemicals of *A. donax* have not yet been identified. Additionally, the effects of *A. donax* infestation on soil microorganisms remain unclear. This information is necessary to understand the invasive mechanisms *A. donax*. *A. donax* is widely cultivated for its economic value. However, its invasive populations have also spread by human activity. There is a need for more concern regarding its invasive traits.

## Figures and Tables

**Table 1:** Invasive mechanisms of *Arundo donax*.

Invasive Characteristics	References
Rapid growth and large biomass production	
⮚ Efficient photosynthetic capacity	[48,49,50,51,52,53]
High vegetative reproduction	
⮚ Ramets from stem and rhizome nodes	[2,31,83]
⮚ Stem and rhizome fragments that contain at least one node	[16,31,87,88,89,90]
High morphological and physiological adaptation to adverse conditions	
⮚ Wide range of habitats	[2,95,96,97,98]
⮚ Cold temperature: Sugar accumulations, ramet sprouting after dieback	[2,22,31,91,97,99]
⮚ Drought: High water-use efficiency, adjustment of osmotic potential, ROS protection	[102,103,105,106,108,111,112]
⮚ Flooding: Aerenchyma formation, anaerobic respiration	[113,114,120]
⮚ Salinity: Adjustment of osmotic potential, ROS protection	[102,126,127,128,129,130,131,133]
High defense ability against biotic stressors	
⮚ Production of toxic substances against pathogens and herbivores	[149,154,155,169]
⮚ Phytolith accumulation against pathogens and herbivores	[176,177]
⮚ Allelopathy	[197,198]
